# A Radar-Based Opioid Overdose Detection Device for Public Restrooms: Design, Development, and Evaluation Study

**DOI:** 10.2196/51754

**Published:** 2023-10-24

**Authors:** Jessica Oreskovic, Jaycee Kaufman, Anirudh Thommandram, Yan Fossat

**Affiliations:** 1 Klick Labs Klick Inc Toronto, ON Canada

**Keywords:** 60 GHz radar, opioid overdose, overdose detection, overdose prevention, respiratory depression

## Abstract

**Background:**

The opioid epidemic is a growing crisis worldwide. While many interventions have been put in place to try to protect people from opioid overdoses, they typically rely on the person to take initiative in protecting themselves, requiring forethought, preparation, and action. Respiratory depression or arrest is the mechanism by which opioid overdoses become fatal, but it can be reversed with the timely administration of naloxone.

**Objective:**

In this study, we described the development and validation of an opioid overdose detection radar (ODR), specifically designed for use in public restroom stalls. In-laboratory testing was conducted to validate the noncontact, privacy-preserving device against a respiration belt and to determine the accuracy and reliability of the device.

**Methods:**

We used an ODR system with a high-frequency pulsed coherent radar sensor and a Raspberry Pi (Raspberry Pi Ltd), combining advanced technology with a compact and cost-effective setup to monitor respiration and detect opioid overdoses. To determine the optimal position for the ODR within the confined space of a restroom stall, iterative testing was conducted, considering the radar’s bounded capture area and the limitations imposed by the stall’s dimensions and layout. By adjusting the orientation of the ODR, we were able to identify the most effective placement where the device reliably tracked respiration in a number of expected positions. Experiments used a mock restroom stall setup that adhered to building code regulations, creating a controlled environment while maintaining the authenticity of a public restroom stall. By simulating different body positions commonly associated with opioid overdoses, the ODR’s ability to accurately track respiration in various scenarios was assessed. To determine the accuracy of the ODR, testing was performed using a respiration belt as a reference. The radar measurements were compared with those obtained from the belt in experiments where participants were seated upright and slumped over.

**Results:**

The results demonstrated favorable agreement between the radar and belt measurements, with an overall mean error in respiration cycle duration of 0.0072 (SD 0.54) seconds for all recorded respiration cycles (N=204). During the simulated overdose experiments where participants were slumped over, the ODR successfully tracked respiration with a mean period difference of 0.0091 (SD 0.62) seconds compared with the reference data.

**Conclusions:**

The findings suggest that the ODR has the potential to detect significant deviations in respiration patterns that may indicate an opioid overdose event. The success of the ODR in these experiments indicates the device should be further developed and implemented to enhance safety and emergency response measures in public restrooms. However, additional validation is required for unhealthy opioid-influenced respiratory patterns to guarantee the ODR’s effectiveness in real-world overdose situations.

## Introduction

The opioid crisis is a growing problem worldwide, with devastating consequences. In the United States alone, fatal overdoses involving opioids claimed the lives of over 80,000 people in 2021 [1]. This problem has been escalating since the 1990s and has seen a fourfold increase from 2010 to 2021, with the rate showing no sign of abating [1]. The more recent surge in opioid-related mortality is largely a result of the rising prevalence of synthetic opioids such as fentanyl, which are substantially more potent than natural opiates [2-4]. Fentanyl and fentanyl analogs are found to be 50-10,000 times more potent than morphine [2,4,5]. These synthetic opioids are added to street drugs without the user’s knowledge to enhance the effects of the drug [6]. As a result, individuals who consume drugs laced with synthetic opioids are at a significantly greater risk of overdosing, as their usual dose may have an unexpected and dangerously high potency [5,6].

The principal mechanism by which opioid overdoses become fatal is respiratory depression through suppression of the region of the brain responsible for respiration rhythm regulation [7], referred to as opioid-induced respiratory depression (OIRD). In the early stages of an overdose, individuals lose consciousness and experience slowed respiration, which can lead to dangerously low blood oxygen levels [7]. Consequently, the individual will experience hypoxia, hypercapnia, and, if left untreated, complete respiration cessation and suffocation [7,8].

Fortunately, there is a fast-acting opioid antagonist called naloxone that reverses the effects of opioid toxicity. Naloxone has a stronger affinity to bond with the same receptor sites in the brain than opioids, thereby blocking the effects of opioids and restoring proper respiration functionality [9-11]. While the effect of naloxone is temporary, lasting around 30-90 minutes, it provides sufficient time for emergency medical personnel to be contacted, arrive, and assume treatment [9-11]. To administer naloxone and save a survivor’s life, the early detection of an overdose is imperative before OIRD has caused blood oxygen levels to drop significantly. When the brain is deprived of oxygen for 3 minutes, consciousness is lost, and lasting brain damage is to be expected [12]. After 10 minutes of oxygen deprivation, if the individual is still alive, they will likely be in a coma and experience permanent brain damage [12]. Therefore, it is crucial that the overdose survivor be discovered as soon as possible to administer naloxone and restore respiration and oxygen flow to the brain.

Many initiatives have been introduced with the goal of improving safety surrounding drug use, such as digital overdose monitoring services, syringe services programs, and supervised injection facilities or safe consumption sites. Digital overdose monitoring services include phone lines where a person can remain on the phone with someone informed of their location and other pertinent information while they use drugs [13]. If the person using drugs becomes unresponsive, the volunteer on the line can contact emergency services and provide the overdose survivor’s location. Syringe services programs supply people who inject drugs with sterile injection supplies to prevent needle reuse and inhibit the transmission of diseases such as HIV and hepatitis [14]. Supervised injection facilities supply trained medical supervision while people use drugs, providing a safe and sterile environment equipped with overdose prevention measures such as naloxone [14,15]. While these safe injection facilities improve safety for people who use drugs and choose to take advantage of them, many people prefer to use drugs in more private settings, such as their homes, or in public facilities, such as restrooms [16]. An exploratory study was completed in New York where business managers were interviewed, and 58% of those questioned had encountered drug use in the restroom of their place of work, indicating the significance of the public restroom drug use problem [17].

The prevention of opioid overdoses in public restrooms has become an increasingly important focus of research and development in response to the opioid crisis [17]. While some businesses have used methods to deter drug use, such as the use of blue lights in restrooms to make veins less visible, restricting restroom access, and even removing stall doors, others have implemented strategies to enhance the safety of individuals who use drugs [17]. For example, supplying naloxone in restrooms and the implementation of antimotion detectors have been proposed as potential solutions [17]. However, while antimotion detectors such as the Brave Sensor or the South End Clinic Anti-Motion Sensor have shown promise in detecting potential overdoses, their lack of specificity means they are prone to triggering many false positives [18,19]. To address this issue, a more precise detection method that monitors respiration, specifically detecting OIRD, could be implemented to enhance opioid overdose identification accuracy and alert staff. This will ultimately produce a more rapid and effective response, as required to reverse overdoses [20].

Respiration monitoring is a customary practice in medical settings and is done using capnography, a medical monitoring technology that uses infrared light to measure the carbon dioxide concentration in expired breath [21]. The capnograph outputs a waveform of carbon dioxide concentration over time, which is representative of inspiration and expiration periods used to calculate respiration rate [21]. If the patient does not require assistance breathing, capnography can be measured with nasal cannulas, or if they are on a ventilator, an adaptor is added to the mouthpiece [21]. While this method has high accuracy in respiration rate monitoring because it tracks respiration waveforms, it requires specialized and expensive equipment [21]. Furthermore, it needs contact with the participant and therefore is not suitable for monitoring in a public restroom.

A simplified means of measuring respiration rate during physical activity is through the use of a respiration belt. The nonelastic belt is affixed around the participant’s thorax or abdomen and uses a strain sensor to detect the corresponding expansion and contraction of the chest wall during respiration. The sensitive strain gauges in the respiration belts allow them to work well to monitor all levels of respiration if they are tightly worn, including measuring OIRD [22]. Although respiration belts do not offer insight into breath composition as provided by capnography, they provide valuable information regarding respiration rate, depth, and patterns. The noninvasive nature of respiration belts makes them a convenient and appropriate solution for a diverse range of applications; however, the belt still requires contact with the participant to monitor respiration.

To identify OIRD in a public restroom setting, a device only needs to measure respiration rate, not breath composition. However, it must be practical, have sufficient precision and sensitivity, and most importantly, be noncontact. Some systems cover most of these requirements but cause concerns for privacy, such as computer vision technologies that can monitor respiration from pixel intensity between frames [23]. Others preserve privacy but are not noncontact, such as pulse oximetry and capnography, which track respiration with a finger monitor or nasal cannulas [23]. A novel noncontact and privacy-preserving smartphone app has been developed for monitoring respiration rates during opioid use and generating alerts in the event of dangerously low respiration rates [24]. This innovative tool leverages short-wave active sonar technology combined with frequency shifts to enhance the sensitivity of the measurement system [24]. This app allows for remote supervision of opioid use in any setting, with the user positioning their phone to face their chest and the app tracking respiration rate by measuring the distance to the user’s chest. The smartphone app requires users to be in a setting where they can position their phone properly and requires a sufficiently charged smartphone. According to a study regarding people who use substances’ acceptability of technological solutions, less than 50% of the people interviewed owned a cellphone [25]. Of those who had a smartphone and access to the internet, less than 70% would consider using it for monitoring their drug use [25]. Most critically, this approach requires active participation from the person using drugs. While this app has the potential to enhance safety during opioid use in any location, like safe injection facilities, it requires forethought and does not address the needs of individuals who are not actively engaged in taking responsibility for their own safety.

We developed a novel approach for detecting and preventing public opioid overdose fatalities. A stand-alone radar device is to be installed in a public restroom stall that can monitor the respiration rate of individuals and subsequently trigger an alert for bystanders to administer naloxone when overdose respiration patterns are detected. The design and development of this device involved several challenges, particularly with respect to ensuring the privacy and anonymity of the individuals being monitored. To overcome these challenges, pulsed coherent radar technology is proposed as a viable, privacy-preserving solution. This compact, low-cost, and high-precision modality allows for the monitoring of respiration rates by detecting changes in the direction of chest movement. The pulsed coherent radar sensor works by emitting a series of high-frequency pulses sweeping through a bandwidth around a set center frequency [26]. The pulses are reflected by body tissues back to the receiver, creating a signal to be analyzed by the system [26]. This technique is derived from the Doppler shift principle, which measures the change in frequency of the received signal caused by chest motion. This technology also controls the phase of the transmitted and received signals, allowing for high-accuracy measurements and extreme sensitivity to very small movements [26]. Because the sensor only records distances, it protects the participant’s privacy. Another benefit of the radar sensor is that the radio waves only reflect off materials with high reflectivity, such as metal, water, and human tissue. This feature allows the radar to sense chest movement and monitor breathing through clothing, even heavy winter coats, making it a viable rescue-alerting solution for many scenarios where a person experiences opioid toxicity in a public restroom.

This paper details the development and preliminary exploratory testing of a radar-based opioid overdose detection device for public restrooms. We provide information about the design process, the respiration tracking ability of the sensor and algorithm, the device’s accuracy in a confined restroom stall and its optimal position, and the device’s ability to monitor respiration during a simulated opioid overdose. The aim of this study is to assess the feasibility of an overdose detection radar (ODR) for use in public restrooms.

## Methods

### Overview

The ODR development was informed by a thorough review of relevant literature and consultation with medical practitioners. Through interviews with emergency medical responders and emergency department physicians, we gained valuable insight regarding the appropriate respiration rate thresholds at which to trigger an opioid overdose alert. We also learned details about the expected position, respiratory condition, and sequence of events that happen to a participant experiencing an opioid overdose. The accuracy of the respiration rate measured by the ODR was assessed in this study.

### Design

Minimizing the size of the device was important to consider throughout the component selection and design process, as the intended use of the device is for small public restroom stalls.

#### Technology Selection

The selection of the Raspberry Pi Zero 2 W (Raspberry Pi Ltd) and Acconeer’s A111 pulsed coherent radar breakout sensor (SparkFun Electronics) for monitoring respiration was based on their performance specifications and compact size. During the exploration phase, the specifications and software development kits for several radar sensors with operating frequencies of both 24 GHz and 60 GHz were compared. The Acconeer A111 60GHz pulsed coherent radar sensor was selected for this application because of its robust software development kit, configurability for the application, and high measurement accuracy, with the 60GHz allowing for the sensitivity and resolution to measure very small movements.

#### Form Factor and Other Considerations

**Figure 1:**
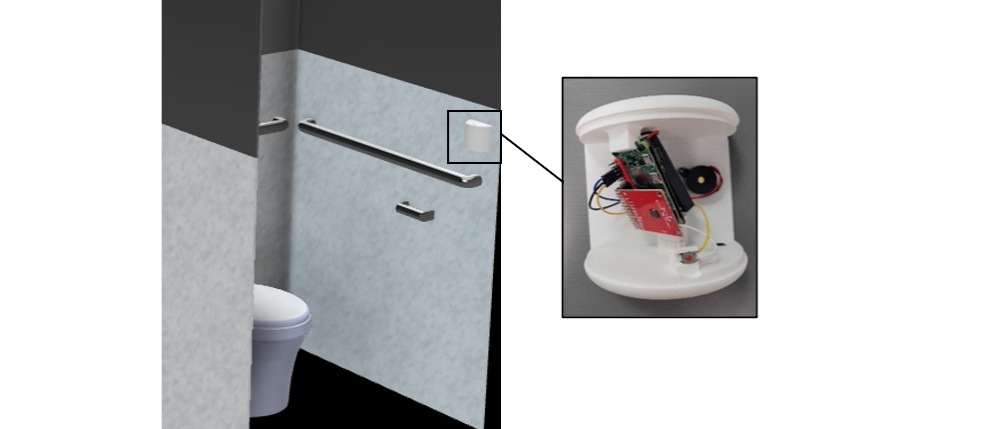
Image of the enclosure and internal components, consisting of the Raspberry Pi Zero 2W, Acconeer’s A111 sensor, and an uninterruptible power supply battery package. For prototyping, the enclosure includes different mounting angles for the electronics. Iterative testing found that the 45-degree angle gave more consistent and accurate measurements.

The enclosure for the ODR was carefully designed to ensure it was discreet and unobtrusive, resembling common restroom fixtures such as air fresheners and smoke alarms (Figure 1). The device was intentionally crafted to not display any visible lights or lenses to avoid concern about identifying information being recorded. While the device does collect and store some data as required for the radar technology, it only records distance data to monitor respiration. The data involved are similar to that used by an automatic flush sensor or motion sensor for lights; therefore, we expect no issues with installing such technology in public restrooms. A key consideration in the enclosure design was its suitability for public settings, requiring it to be tamper-resistant, durable, easy to clean, and securely mountable to restroom stall walls. For this, we designed a compact (10 cm × 11 cm × 6 cm) hemicylindrical enclosure. The enclosure is currently 3D-printed with polylactic acid, a durable and cost-effective material. Further iterations may progress to an injection-molded design.

### Position

According to the sensor specifications, the optimal position for the ODR is directly facing the participant’s chest within the programmed range. However, it was found through consultation with professionals that the device must be designed with consideration that the individual will likely lose consciousness before their respiration rate drops to dangerous levels. Thus, the device must accurately measure respiration when the person is slumped in all different directions within the stall. Due to the bounded capture area of the radar, 65 degrees horizontal and 53 degrees vertical, it was difficult to monitor the entire volume of the stall. Therefore, the scope was limited to scenarios where the participant was slumped over while remaining on the toilet seat. In-laboratory experiments were conducted using a mock restroom stall setup following building standards for public restroom stalls: 890-940 mm wide, 1500 mm long, and with an 860 mm door opening [27].

**Figure 2:**
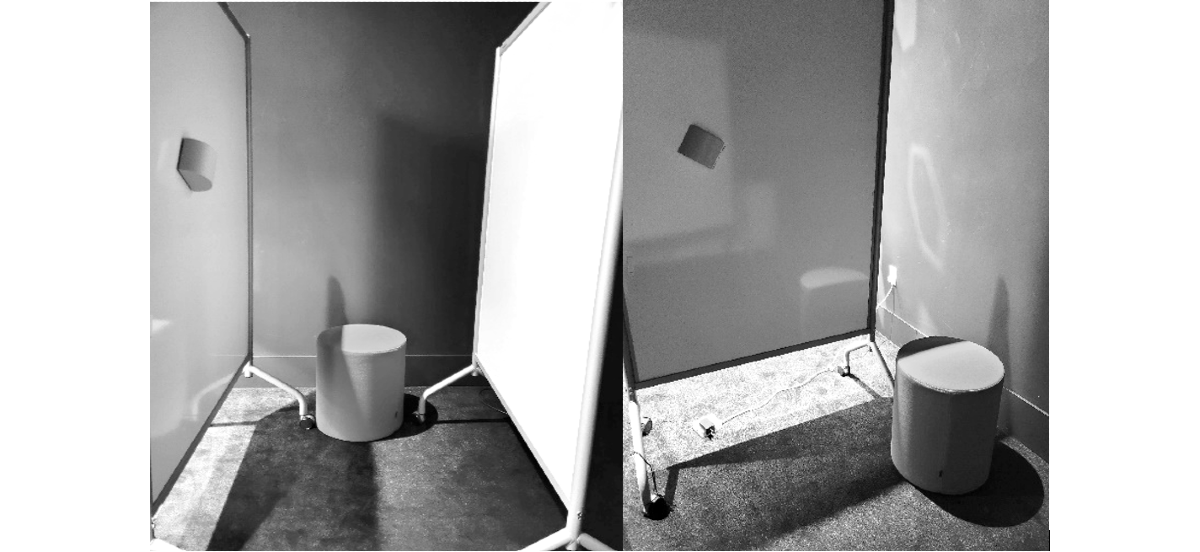
Mock restroom stall setup according to building code regulations (890-940 mm wide and 1500 mm long), with the toilet centered between the side walls.

The participant was seated in the restroom stall setup (Figure 2) with the ODR attached to the wall at different distances, heights, and angles from the participant’s chest. The device was secured to the side wall using Velcro strips, allowing for easy repositioning. To determine an optimal position, the targeted respiration rate was held constant at 15 breaths per minute (bpm) by following audible cues to inhale and exhale. Once the device accurately detected respiration while the participant was seated upright, the participant slumped in various directions to ensure respiration could be tracked accurately in different body positions. The measured respiration rate was monitored to ensure it continued to accurately measure approximately 15 bpm while the participant slumped in various directions to approximate an overdose. If the device failed to track respiration while the participant was slumped in any direction, the device position was adjusted, and the experiment was repeated until respiration was consistently tracked in all slumped positions.

### Respiration

The respiration tracking algorithm implemented for opioid overdose monitoring is a customized version of Acconeer’s A111 SDK breathing module. Acconeer’s Python algorithm uses distance measurements to monitor the respiratory cycle by tracking the relative distance of the reflecting body [28]. An exhale is registered when the distance of the reflecting body increases, indicating that the chest surface is moving away from the sensor, while an inhale is registered when the distance decreases, indicating that the chest is moving toward the sensor. To avoid incorporating body movements into the respiration tracking, the algorithm imposes constraints on the distance of the motion used to calculate respiration, limiting it to a range between 0.75 mm and 20 mm. Each breath cycle is defined by 1 inhale and 1 exhale, which are identified based on the change in distance. The duration of a breath cycle is subsequently used to compute the current respiration rate. The algorithm accounts for variations in respiratory patterns that may lead to brief inaccuracies in the calculated respiration rate. To customize the device for the opioid overdose detection application, we added respiration rate thresholds and respiration cessation triggers for detecting breathing patterns consistent with opioid overdoses.

Respiration validation was completed for 2 positions. The first validation testing protocol for the device included a healthy participant outfitted with the Go Direct respiration belt (Vernier) and seated upright in a chair directly in front of the radar device at a distance of 1.5 m. The participant was instructed to breathe at a comfortable pace for approximately 5 seconds until both measurement devices began recording, and then to hold their breath for 3 seconds. This breath-hold event was included to allow the data sources to be synced in analysis and remove any delays caused by setup or calibration time after the algorithm was started.

The second respiration validation protocol was modified to include the radar in its final position. This was completed to ensure that respiration measurements were accurate for multiple participants. Participants were outfitted with the Go Direct respiration belt for validation purposes and followed the same protocol as previously described. Each participant recorded 2 tests with the device fixed in the mock stall in its mounting position and the participant seated upright on the mock toilet. Breathing motion captured from the radar was compared with the peaks recorded from the respiration belt, and the error between respiration periods was computed.

OIRD is a life-threatening condition characterized by a significant decrease in breathing rate caused by opioids. To establish a reliable threshold for triggering an overdose alert, consultation was sought from medical professionals. According to medical experts and literature, a sustained respiration rate of less than 8 bpm is considered dangerous and requires immediate intervention [29]. Accordingly, the overdose detection algorithm has been designed to trigger an alert when the respiration rate falls below this threshold for 15 seconds or 2 full breaths. The inclusion of 2 breaths enables the algorithm to discern a momentary low respiration rate reading caused by intentional motion from an actual overdose event, including a sustained lowered rate. Additionally, the algorithm is programmed to trigger an alert if no respiration motion is detected for 10 seconds, providing an added layer of protection if respiration drops too quickly to track.

The duration of the respiration period is crucial in computing the respiration rate. Therefore, we elected to assess the error by analyzing the mean difference between the respiration periods calculated from the respiration belt and the overdose radar. The direction of the error was included because it is important to know whether the respiration rate over several breath cycles will remain accurate, regardless of an error in a single respiration cycle duration.

### Overdose Simulation

Validation testing was performed on the ODR’s ability to monitor respiration rate in the mock stall while the participants simulated an overdose by slumping in all directions. The protocol for this validation test includes outfitting the participant with the respiration belt, sitting upright in the mock stall, and breathing at a comfortable pace for 30 seconds. The participant is then signaled to simulate falling unconscious by slumping forward, then backward, left, and right, holding each position for 30 seconds and breathing at a comfortable pace. The ODR’s respiration tracking algorithm and the respiration belt recorded data throughout all positions and movements, and the data was analyzed to determine the agreement between the algorithm and belt respiration periods in each position.

### Ethical Considerations

Because of the challenges of running a study in a public restroom, including privacy and health concerns, to confirm the performance of the ODR, preliminary testing was conducted in-laboratory on 3 of the authors (JO, JK, and YF), who are all healthy and consented to perform validation tests and approximate an overdose scenario in a mock restroom stall. All the data recorded in the preliminary testing contained no identifying information and only recorded respiration patterns. To ensure the safety of the human participants, the ODR was designed using approved, commercially available devices, and any testing was done to help inform the development of the device. Therefore, as per Article 6.11 of the Tri-Council Policy Statement on Ethical Conduct for Research Involving Humans, we did not require approval from the Research Ethics Board for this exploratory testing [30]. Additionally, the testing procedures did not require the participants to perform any potentially dangerous respiration patterns to replicate an opioid overdose. Participants were able to breathe at a self-selected, comfortable pace throughout all experiments, and while the radar is unaffected by what type of clothing is worn, participants wore light to medium-weight long-sleeved clothing for the described tests.

## Results

### Overview

The experimental procedures outlined in this study involved the use of 2 distinct data sources: the respiration belt, which measures force in Newtons, and the radar, which measures distance in millimeters. Although the sources provide differing data signals, both can provide valuable information on breath timing and can be used to calculate the duration of each respiration cycle or period. We retained the sign of the difference instead of studying the absolute difference since a slight deviation in the peak could yield 1 positive and 1 negative difference while still resulting in the same respiration rate. The respiration period is important in the context of the overdose detection algorithm, where the respiration rate is a critical feature.

### Position

**Figure 3:**
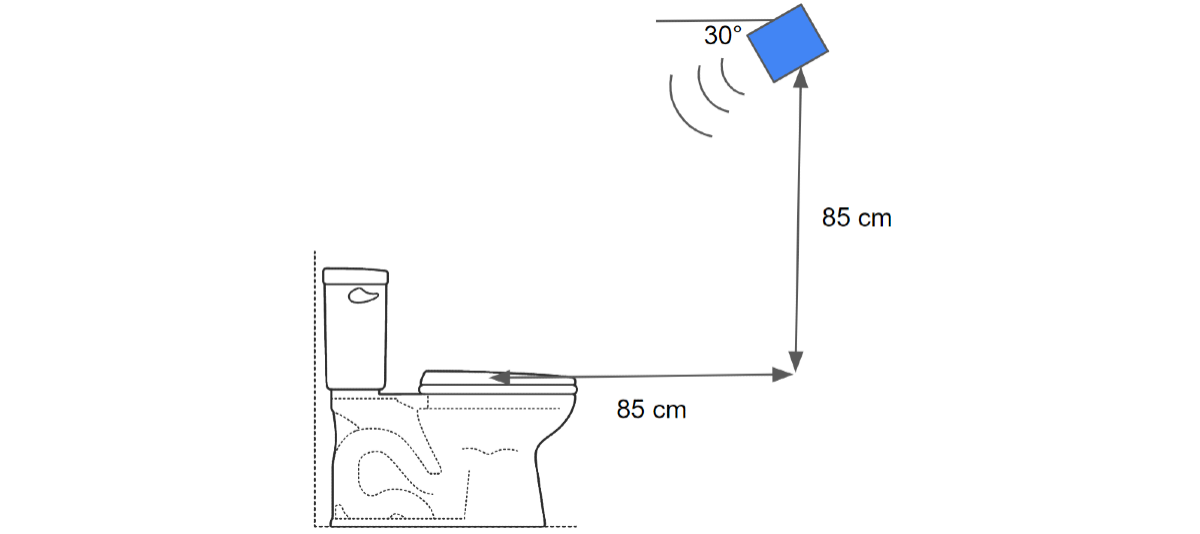
Radar positioning. The optimal position for the radar device in the mock restroom stall was found to be 85 cm in front of the center of the seat, 85 cm above the top of the seat, and angled 30 degrees down toward the toilet. Additionally, inside the casing, the sensor was angled at 45 degrees out from the wall to target the participant’s chest while seated on the toilet. Through testing, this position was capable of consistently monitoring respiration with the participant in many different slumped positions.

The initial placement of the radar device was at chest height, tilted at an angle of 45 degrees away from the wall, directed toward the chest. The sensor-chest distance was situated within the range of 0.75-1.5 m. However, when the participant slumped forward, the radar was aimed directly at the top of the head, which did not display any motion related to respiration. To address this limitation, we iteratively adjusted the radar height and tilt angle to target the chest directly. Eventually, the optimal position was determined to be 85 cm horizontally from the front of the chest (center of seat) while seated upright, 85 cm above the top of the toilet seat, angled 30 degrees down from horizontal, and at a 45-degree angle setting within the device pointing out from the wall toward the toilet (Figure 3). The 30-degree downward angle facilitated a direct path toward 35 cm above the toilet seat, specifically targeting the chest and abdomen at a distance of approximately 1 m. This elevated position allows the chest to be monitored when the participant is upright or slumped backward and the back to be monitored if the participant collapses forward.

### Respiration

**Figure 4:**
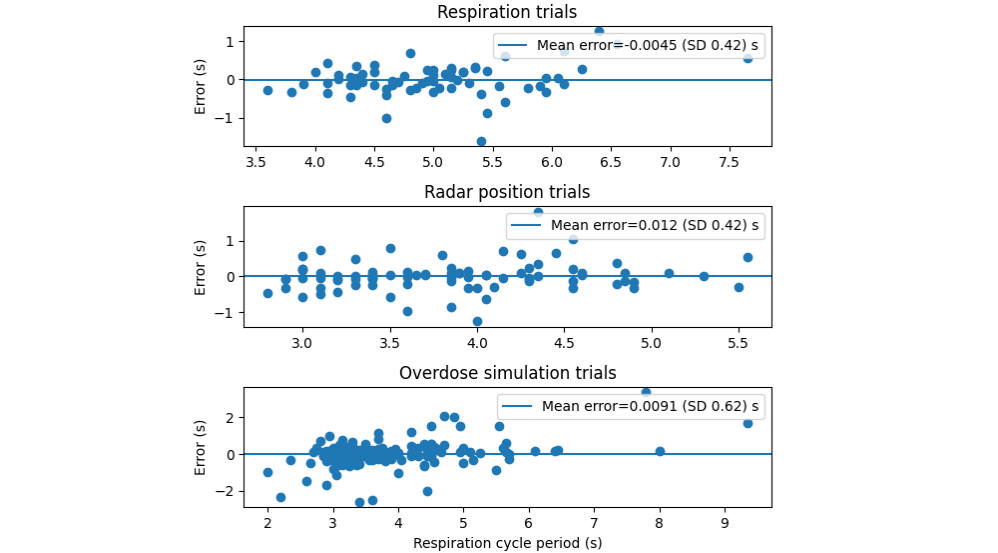
Errors in measurement between the radar and the respiration belt for the duration of each respiration cycle monitored were split into each of the test protocols. The mean error for all tests is minimal, with similar SDs between tests. The SD is larger as it demonstrates that the error in cycle duration can be positive or negative, but overall, it balances out to a very small mean error, having a small effect on the respiration rate over time.

**Figure 5:**
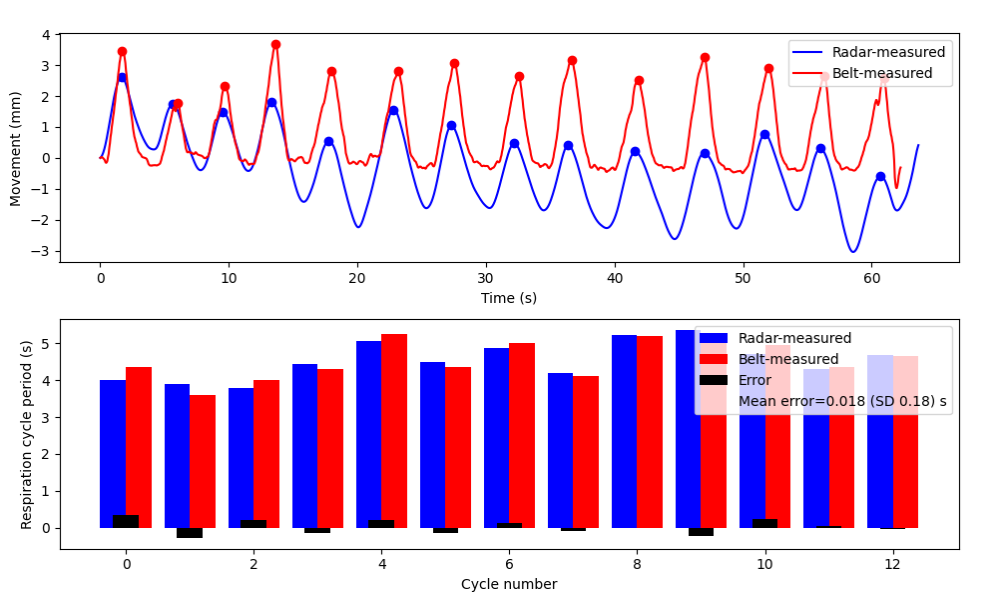
Representative respiration experiment: breathing motion captured by overdose radar (blue) and respiration belt (red) in the first plot. The participant was breathing comfortably while seated upright, directly 1.5 m in front of the radar sensor. The second plot shows the respiration cycle period in seconds calculated from peak-to-peak distance for both radar-measured (blue) and belt-measured (red) breathing movements. The error is shown in black, and the mean error is 0.018 (SD 0.18) seconds.

In the initial benchtop validation experiment, a participant was seated upright in a comfortable position while their respiration was recorded simultaneously by the respiration belt and radar. A total of 67 respiration periods were analyzed over 6 experiments (2 per participant). These tests revealed a favorable agreement in peak distance between the radar and the respiration belt, with a mean error in cycle duration of 0.0045 (SD 0.42) seconds (Figure 4; representative test displayed in Figure 5).

**Figure 6:**
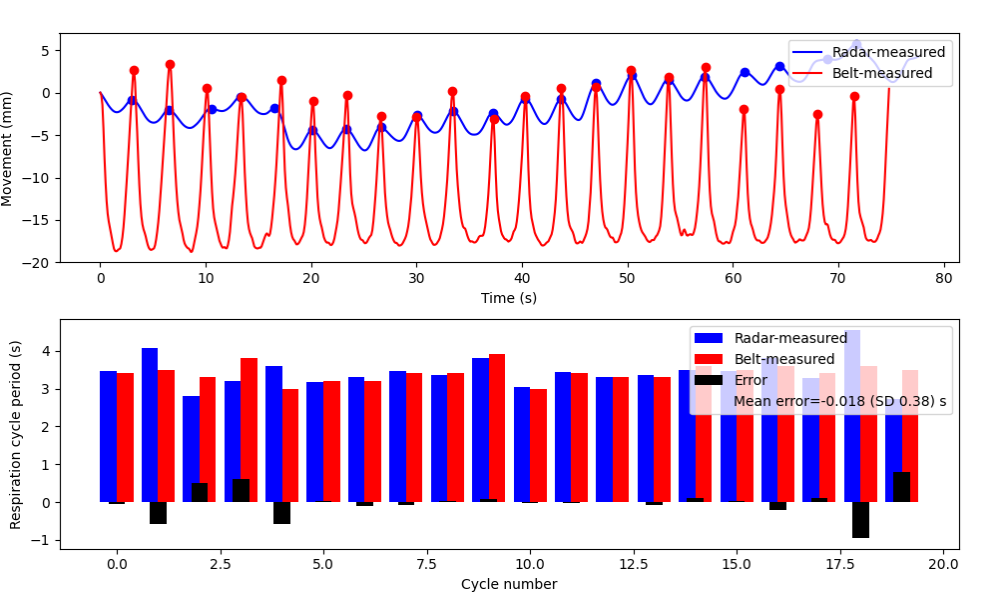
Representative position experiment: the top plot shows breathing motion captured by overdose radar (blue) and respiration belt (red). The participant was seated upright in the mock restroom stall with the sensor mounted at 85 cm forward, 85 cm above, and angled 30 degrees down toward the toilet seat. The second plot shows the respiration cycle period in seconds calculated from peak-to-peak distance for both radar-measured (blue) and belt-measured (red) breathing movements. The error is shown in black, and the mean error for the test is –0.018 (SD 0.38) seconds.

A secondary set of validation tests was conducted to evaluate the tracking accuracy of the radar sensor’s position in detecting respiration. Participants were seated upright in the mock stall, with the radar device placed in its optimal position. Participants were instructed to breathe at a comfortable pace, and their breath periods were measured and compared using both the respiration belt and radar methods. A total of 85 respiration cycles were analyzed for the position validation testing, and good agreement was observed across all tests (Figure 4). The mean breath period error was found to be 0.012 (SD 0.42) seconds, and a representative test is presented in Figure 6.

### Overdose Simulation

**Figure 7:**
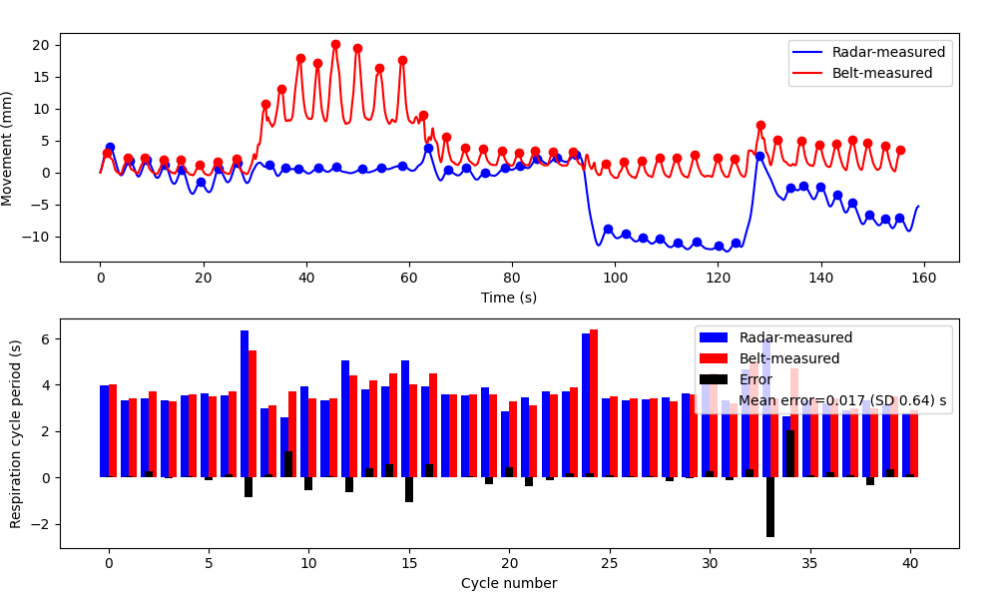
Representative overdose simulation experiment: the top plot shows breathing motion captured by overdose radar (blue) and respiration belt (red). The participant was seated upright in the mock restroom stall for 30 seconds before slumping forward, backward, left, and right, holding each position, and breathing comfortably for 30 seconds. The bottom plot shows the respiration cycle period in seconds calculated from peak-to-peak distance for both radar-measured (blue) and belt-measured (red) breathing movements. The error is shown in black, and the mean error for the test is 0.017 (SD 0.64) seconds. This signifies that while the cycle errors can vary positively and negatively, they balance out to a small mean error of 0.017 seconds, showing that the cycle error has a minimal effect on calculated respiration rate over time.

In the final evaluation, the radar position was evaluated under simulated overdose scenarios by instructing participants to slump over in various directions while wearing the respiration belt for validation. The radar device was capable of tracking respiration even as participants transitioned into different slumped positions, causing much shallower respiration with minimal calibration delay, as demonstrated in Figure 7. Across all overdose simulation tests, which encompassed a total of 204 respiration cycles, the respiration was effectively tracked, with a mean period difference of 0.0091 (SD 0.62) seconds (Figure 4). It is worth noting that the slightly larger error can be attributed to the inherent noise present in the radar signal during positional changes, which was anticipated.

Overall, after analyzing the error for all respiration cycles, including all 3 protocols, the error was 0.0072 (SD 0.54) seconds.

## Discussion

### Overview

With further development and tuning, the ODR has the potential to detect opioid overdoses in public restrooms with accurate respiration tracking, finding a mean error of 0.0091 (SD 0.62) seconds when monitoring respiration cycle duration in overdose simulation experiments. This study has demonstrated that respiration can be accurately monitored within a restroom stall using a pulsed coherent radar sensor and a Raspberry Pi. The identification of OIRD could be used to alert staff or bystanders to the emergency and mobilize a rescue response.

### Principal Findings

Following an iterative process to ensure monitoring of all slumped positions, the final device mounting position for the ODR was determined to be on the side wall of the stall. The device was mounted 85 cm above the toilet seat and 85 cm in front of the surface of the chest or center of the seat, angled down by 30 degrees. While concerns were initially raised regarding the placement of the device in locations that may not be feasible for all public restrooms, regulations for larger handicap stalls to have support bars next to the toilet on the adjacent wall alleviated these concerns as it meant that toilets would always be a standard distance from the wall [27].

The benchtop validation experiments demonstrated the capability of the radar to monitor respiration with accuracy and consistency in controlled settings. The signal processing involved in the A111 breathing algorithm might cause slight variations in the duration of the respiration cycle, as filtering the signal could lead to flattening of the peak, which may cause offset peak locations. Nevertheless, given that the respiration rate is a crucial feature for detecting overdoses, the slight shift in peak location would not significantly impact the calculated respiration rate as it would be counterbalanced by a negative shift in the adjacent period rather than accumulate drift.

The peak-to-peak distances are important, but the waveform morphology and peak magnitudes were disregarded because each method measures distinct physiological data; the radar measures the distance to the chest in millimeters, while the respiration belt gauges the external force exerted by the chest wall in Newtons. Therefore, in this preliminary testing, the peak distances measured by the radar were compared with the peak distances recorded by the respiration belt.

Exploratory validation testing was conducted to compare ODR motion-derived respiration periods with those collected using a respiration belt. Good agreement between the 2 methods was observed for both benchtop testing with the participant seated upright directly in front of the sensor as well as in a mock restroom setup with the ODR mounted to the wall. Minimal deviations in respiratory cycle duration were observed. The variance in error is due to single peaks being shifted, resulting in positive and negative errors in adjacent respiration cycles. While this error is important to identify, it does not significantly impact the overdose detection algorithm’s analysis of respiration rate, as the errors are not sustained for a window long enough to affect the algorithm’s functioning (15 seconds).

The principal source of error in the overdose simulation experiments, as seen in Figure 7, was related to participant movements changing to a different slumped position. It is worth noting that the device uses relative movement tracking to monitor respiration, requiring recalibration to ensure accurate tracking across different positions. The study participants encountered difficulty assuming a stable slumped position immediately after moving and often resorted to readjusting for 1-2 seconds before settling into a stable position that could be maintained for the 30-second test period. This poses a challenge in differentiating whether the radar’s recovery time after each position change is delayed due to its calibration process or because the participant is still in motion. While the device’s loss of respiration tracking during large movements was expected, it was able to rapidly restore accurate tracking.

### Limitations and Future Work

This preliminary testing included limitations that will be considered in the design of a future pilot study conducted on a larger and more diverse sample size. First, the overdose simulation experiments were not representative of a fatal opioid overdose. Second, while the device does have a battery backup, it currently relies on being connected to a power supply. Third, the device’s field of view, while tuned well for a restroom stall, would not accommodate a larger-than-standard space. Finally, we have not yet consulted with people involved in harm reduction or people who use drugs regarding the details of the implementation and alert system. We have considered how these limitations will be addressed moving forward.

The overdose simulation tests did not replicate an actual opioid overdose scenario because healthy participants found it strenuous to repeat the respiration patterns observed during fatal overdoses. Instead, the participants were instructed to breathe at a comfortable pace during the experiments. This was beneficial as they were able to breathe shallowly rather than gasping for large breaths to maintain a slow respiration rate. The shallow breathing was beneficial to include in the study because it confirmed that the radar had a high sensitivity to respiration movement, accurately measuring even very weak respiration activity. Because the preliminary testing conducted used healthy participants breathing normally when the device’s primary objective is to capture irregular and unhealthy respiration patterns, a future study is planned to validate the device on unhealthy patients. In the pilot study, unhealthy respiratory patterns will be monitored by the ODR and compared against the gold standard respiratory tracking output.

The next consideration to be addressed is the power supply of the device. For these experiments, the ODR was connected to a power supply using a USB cord and included a battery backup. To facilitate the device’s implementation and use, it will be modified to be battery-powered. The high-sensitivity measurements and on-board processing required to calculate respiration rates consume a considerable amount of battery power; therefore, we plan to integrate a less power-intensive presence detection component into the algorithm that triggers respiration monitoring upon detecting human entry into the restroom stall. Once an opioid overdose is detected during respiration monitoring, the device will temporarily switch back to presence detection to verify human presence before alerting. This feature optimizes battery life and reduces false positives that could occur when the device continues to track respiration after a person has left the stall.

Because this device was designed to monitor a standard public restroom stall, the position and field of view were selected accordingly. A limitation of this device’s position in a larger stall is that individuals may collapse into an open space, fall to the floor, and potentially be out of the detection range. Unfortunately, this scenario cannot currently be accommodated in the device design as the detection range is limited and needs to be centered around where the participant’s chest is when seated upright to capture all other positions. Importantly, the device can measure respiration in all body positions of someone using the toilet, meaning that the device is able to monitor healthy and unhealthy people regardless of their position.

While this study is limited to the development and evaluation of the ODR device, forethought has been considered about its application. We aim to gather insight from businesses and people who use drugs on how to implement this device in a way where we can maximize its life-saving potential. While it would be beneficial to notify patrons of the device’s presence and functionality, it can be understood that business owners may not appreciate advertising their facilities as a safe space for drug use. Conversely, people who use drugs could be deterred by such a device if they are concerned it may result in police presence with the stigma surrounding drug use. Regardless of the mentioned concerns, both groups of people can recognize the device as a life-saving measure that can make a significant impact on the safety of the community.

The technology design is currently limited to implementation in a public restroom stall to detect opioid overdoses. However, other applications could be possible with slight design modifications to change specifications such as the detection area, device position, or battery size. These modifications could allow the continual monitoring of other sites such as addiction treatment facility rooms, hospital rooms, and holding cells.

### Conclusion

This study describes the design, development, and exploratory research of a device with promising potential to monitor respiration rate in a restroom stall setting and detect opioid overdose events. The ODR successfully monitors respiration in a restroom stall setup when the participants are in a variety of slumped positions consistent with overdoses. The development of this opioid ODR considers the physiological aspects of opioid overdoses as recommended by medical professionals, and the device is tailored to accurately monitor respiration, the main indicator of opioid overdose.

The end goal of this device concept is to add an additional layer of safety to public restrooms, enabling the timely administration of naloxone and the mobilization of rescue teams in response to overdose emergencies. Future pilot and research studies will help design the device to detect overdoses in public restroom stalls. Given the rapidly escalating opioid epidemic, it is essential to seek and develop innovative solutions that can help protect the public and facilitate prompt and effective responses to overdose events. The results of this study represent a crucial step forward in this regard, and further investigations are warranted to validate the device’s performance under a variety of conditions and settings, including being evaluated on people who use drugs to ensure we can accurately track the afflicted respiration patterns.

## Abbreviations

bpm: breaths per minute
ODR: opioid overdose detection radar
OIRD: opioid-induced respiratory depression

## Data Availability

The data sets generated during and/or analyzed during this study are available from the corresponding author on reasonable request.
